# Supplementary material for: Cuscutae Japonicae Semen Ameliorates Memory Dysfunction by Rescuing Synaptic Damage in Alzheimer’s Disease Models
Source: Nutrients. 2019 Oct 28;11(11):2591. doi: 10.3390/nu11112591 (PMC6893468; doi:10.3390/nu11112591)
Supplement: Supplementary file 1 [file nutrients-11-02591-s001.zip › Figure S1.pdf]

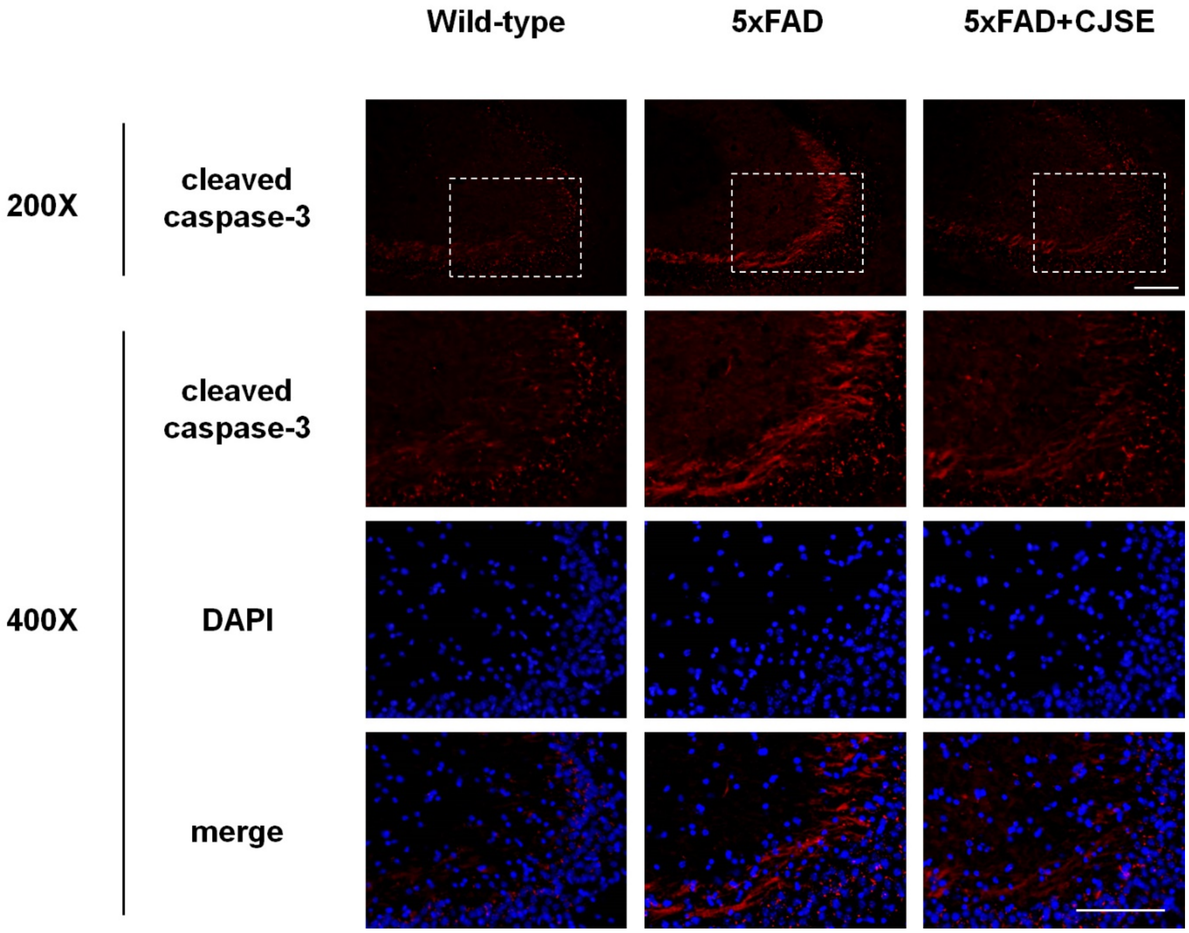

Figure S1. Effects of CJSE on expression of cleaved caspase-3 in 5xFAD mice. Representative photomicrographs cleaved caspase-3 immunoreactive regions in hippocampal CA3 are shown; scale bar=100 μm. CA3; cornu ammonis
