# Supplementary material for: Cuscutae Japonicae Semen Ameliorates Memory Dysfunction by Rescuing Synaptic Damage in Alzheimer’s Disease Models
Source: Nutrients. 2019 Oct 28;11(11):2591. doi: 10.3390/nu11112591 (PMC6893468; doi:10.3390/nu11112591)
Supplement: Supplementary file 1 [file nutrients-11-02591-s001.zip › Figure S2.pdf]

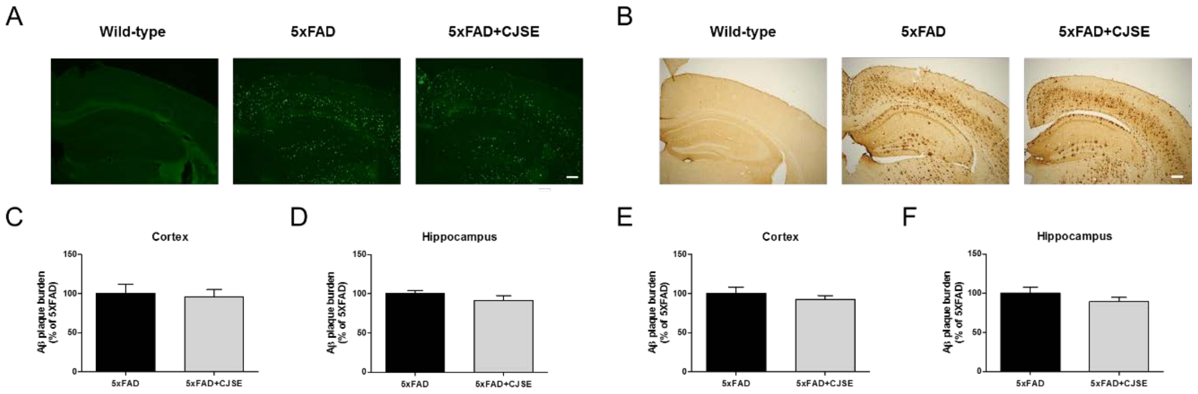

Figure S2. Effects of CJSE on Aβ deposits in 5xFAD mice. (A and B) Representative photomicrographs of thioflavin S staining and immunostaining with 6E10 ($A\beta_{1\text{-}16}$ antibody), respectively; scale bar=200 μm. (C and D) Quantifications of thioflavin S-stained area in cortex and hippocampus, respectively. (E and F) Quantifications of 6E10-immunoreactive area in cortex and hippocampus, respectively. Values are indicated as the mean ± S.E.M.
